# Influence of the use of testosterone associated with physical training on some hematologic and physical parameters in older rats with alloxan-induced diabetes

**DOI:** 10.1590/2359-3997000000200

**Published:** 2016-08-31

**Authors:** Romeu Paulo Martins Silva, Rodrigo Otávio dos Santos, Nelson Eurípedes Matildes, Antônio Vicente Mundim, Mario da Silva Garrote, Pâmella Ferreira Rodrigues, Nilson Penha-Silva

**Affiliations:** 1 Centro de Ciências da Saúde e Desporto Universidade Federal do Acre Rio Branco AC Brasil Centro de Ciências da Saúde e Desporto, Universidade Federal do Acre (UFAC), Rio Branco, AC, Brasil; 2 Centro Universitário do Planalto de Araxá Araxá MG Brasil Centro Universitário do Planalto de Araxá (Uniaraxá), Araxá, MG, Brasil; 3 Instituto de Genética e Bioquímica Universidade Federal de Uberlândia Uberlândia MG Brasil Instituto de Genética e Bioquímica, Universidade Federal de Uberlândia (UFU), Uberlândia, MG, Brasil

**Keywords:** Anabolic steroid, aging, diabetes, physical training

## Abstract

**Objective:**

This study investigated the possible blood changes in wistar rats elderly with and without treatment with anabolic steroids submitted physical training.

**Materials and methods:**

Elderly rats (32) were divided into four groups: normal (N), treated normal (NT), diabetic (D) and treated diabetic (DT). They were submitted to 20 sessions of swimming with overload (5% body weight), 40 min/day for four weeks. The NT and DT groups received application of testosterone twice a week. At the end of the sessions, the animals were subjected to swimming until exhaustion and then killed for removal of blood and visceral fat. We evaluated maximum swim time, weight of visceral fat, erythrogram, leukogram, lipidogram and serum levels of glucose, lactate, aspartate aminotransferase and creatine kinase. The results were compared using one-way ANOVA followed by the post hoc Tukey test.

**Results:**

In elderly diabetic rats, the use of anabolic associated with physical training in older rats resulted in improvement in erythrogram, lipidogram and physical performance for high-intensity aerobic exercise. However, it was related to changes in leukocyte count, probably associated with inflammation.

**Conclusion:**

The combination of the use of testosterone with physical training, followed by maximal effort test caused changes hematological and biochemical can be associated with improvement in physiological characteristics, with increase of the swimming time and decrease of visceral fat levels, improvement in aerobic metabolism of fatty acids and glucose in normal and diabetic animals.

## INTRODUCTION

Diabetes mellitus is characterized by a deficiency in the production or action of insulin, or both, resulting in increased blood glucose in untreated individuals ( 1 ).

Diabetes can be induced in experimental animals by chemical substances such as alloxan or streptozotocin. These substances cause irreversible lesions in the pancreatic cells producing insulin. This causes a large reduction in the production of insulin, which results in diabetes ( 2 ).

The deficiency of insulin causes loss of muscle mass due to exacerbation of the decline of proteolysis and protein anabolism.

Besides insulin, protein anabolism is also stimulated by testosterone, androgen steroid hormone produced by the testes and the adrenal ( 3 ). Testosterone promotes increased mass ( 4 ) and muscle strength ( 5 ).

There is evidence that treatment with these compounds can improve the resistance of skeletal muscle against fatigue, which increases the tolerance of experimental animals to physical activity ( 6 ).

The intense physical exercise can alter the concentration of blood cells and increase the use of glycogen, resulting in glucose fall ( 7 , 8 ) and increased production of lactic acid.

Testosterone can also assist in modulating the body’s immune response ( 9 ), by changing the activity of leukocytes and other cells of defense ( 10 ).

Physical exercise causes numerous physiological and metabolic adjustments in the immediate or long term so that the body can supply a higher energy demand and to remain in homeostasis ( 11 ).

Although it is known that anabolic steroids can increase the tolerance to physical activity ( 6 ), the effects of the combination of many synthetic anabolic steroids with the exercise still need to be better known ( 12 ).

The purpose of this study was to analyze the effects of anabolic steroids on physical, hematological and biochemical variables in diabetic rats under aerobic physical training.

## MATERIALS AND METHODS

The study was conducted in accordance with the recommendations of the Brazilian College of Animal Experimentation (COBEA), with the National Council for Animal Experiments Control (CONCEA) and with the Federal Law guidelines, 11,794, October 8, 2008 – Brazil.

### Induction of diabetes by alloxan

Induction of experimental diabetes mellitus followed the protocols described in literature ( 13 ). After 24 hours of fasting, animals received intravenous injections (tail vein) of alloxan monohydrate (Sigma, St Louis, MO, USA) in 0.01 M citrate buffer, pH 4.5 (35 mg/kg body weight). The control rats underwent similar handling but only with injection of a citrate buffer solution. Two weeks after that treatment, rats that had levels of fasting glucose greater than or equal to 126 mg/dL were considered diabetic and used in the study ( 1 ).

### Experimental groups

For this study we used 32 male albino rats (12 months) from Wistar race ( *Rattus norvegicus* ), distributed randomly into four groups (eight rats in each): normal animals without treatment (N), untreated diabetic animals (D) normal animals with treatment (NT) and diabetic animals with treatment (DT).

### Containment and nutrition

The animals were kept in collective cages (100 x 50 x 30 cm) with an average of 3 rats per cage. The temperature inside the chamber of the vivarium was maintained between 22-25 °C in a room with controlled photoperiod set to 12 h of light and 12 h of darkness. All animals were fed with standard balanced diet and water *ad libitum* .

### Adaptation and physical training

The animals were subjected to an adjustment period of two weeks. During this period, the animals were subjected to physical exercise with initial duration of 10 min. Every three days the duration of exercise increased by 10 min until reach 40 min at the end of two weeks, with rest on Saturdays and Sundays.

After the adjustment period, the animals maintained the 40 min of physical exercise for six weeks, Monday to Friday, between 2 and 5 PM. This period of training was higher than achieved in other studies. The exercises consisted of sessions of swimming with moderate intensity, with 5% body weight tied to the tail. The animals swam in an adapted tank with depth of 48 cm and water temperature maintained between 30 and 36 °C ( 14 ).

### Treatment with anabolic

During the six weeks of physical training, the animals of the NT and DT groups received intramuscular injections of the mixture of esters of testosterone (testosterone propionate, 30 mg; testosterone phenyl propionate, 60 mg; testosterone isocaproate, 60 mg; testosterone decanoate, 100 mg; benzyl alcohol, 0.1 mL; and peanut oil, qs 1 mL) (Durateston™, Organon, Brazil), with syringes of 1 mL (15 mg/kg body weight) twice a week (on Tuesdays and Fridays at 4h30min PM).

Animals not treated with the anabolic (groups N and D) received intramuscular injections of peanut oil ( 15 ).

### Test of maximum effort and sacrifice of animals

At the end of six weeks of training, the animals were subjected to intense physical exercise until exhaustion. It was considered that the exhaustion was achieved when the animal could not keep their noses out of the water for more than 10 s. Once the exhaustion was reached, the animals were removed from the water and placed on a bench. Body and tail were carefully dried with sterile paper towels. After the last session of exercise until exhaustion, the animals were anesthetized with ketamine and xylazine and sacrificed by decapitation in guillotine. Laparotomy was performed to collect the retroperitoneal fat, mesenteric and epididymal in analytical balance.

### Collection of blood samples

The blood samples were taken at the end of six weeks of treatment. Before and after the exhaustion test, samples of 25 µl of blood were transferred from the tail of each animal, using heparinized capillary glass, directly into vials containing 50 µl of 1% sodium fluoride for inhibition of glycolytic activity. These samples were used for determination of glucose and lactate. After the exhaustion test, two blood aliquots were collected by cardiac puncture. The first sample (1 mL), collected in tubes containing K_3_EDTA as anticoagulant, was used to perform the hematologic analyses. The second sample (3 mL), collected in tubes without anticoagulant, was centrifuged at 720 x *g* and the serum was transferred to sealed vials and stored under refrigeration until the time of biochemical analysis.

### Dosage of glucose and lactate

The blood glucose was determined using the Accu-Chek apparatus (Roche, São Paulo, SP, Brazil). The blood lactate was analyzed by electro-enzymatic method in an automatic analyzer (YSI 1500 Sport L-Lactate, USI, Yellow Springs, Ohio, USA).

### Erythrogram

The erythrogram, including the determination of the erythrocyte indices [mean corpuscular volume (MCV), mean corpuscular hemoglobin (MCH) and the mean corpuscular hemoglobin concentration (MCHC)], were performed in automated cell counter ABC Vet (ABX Diagnostics, Montpellier, France) using card specific to rats.

### Leukogram

The leukometry was also carried out in the automatic cell counter ABC Vet (ABX Diagnostics, Montpellier, France). The identification and differential counting of leukocyte were performed with preparations stained by the method May-Grünwald-Giemsa in optical microscope.

### Lipidogram and determination of serum activities of AST and CK

The activities of creatine kinase (CK) and aspartate aminotransferase (AST) and levels of total cholesterol, triacylglycerols (TAG) and cholesterol of high density lipoprotein (HDL-C) were determined by automated methods, using specific commercial kits (Labtest) in automatic multichannel analyzer (ChemWell, USA) previously calibrated and measured with calipers (Calibra 1H) and control serum (Qualitrol 1H). The cholesterol of lipoprotein, low-(LDL-C) was calculated using the equation LDL-C = total cholesterol - (HDL + TAG / 5) ( 16 ).

### Statistical analysis

The results between the different groups were compared with use of ANOVA, with *post hoc* Tukey test, and were considered significantly different when *p* < 0.05. The tests were conducted using the application GraphPad Prism 5 (GraphPad Software, San Diego, CA, USA).

## RESULTS

Table 1 presents the average values obtained in the erythrogram of different groups. The concentration of hemoglobin, the red cell count and hematocrit values were higher in treated groups NT and DT in relation to non-treated groups N and D ( *p* < 0.05). But there was no difference in these parameters between diabetic and normal groups in the absence and in presence of treatment with anabolic ( *p* > 0.05). The mean corpuscular volume (MCV) was also higher in treated groups (NT and DT) than in groups that were not treated with testosterone (N and D), being higher in DT group than in NT ( *p* < 0.05). The mean corpuscular hemoglobin (MCH) was higher in treated groups (NT and DT) than in group N ( *p* < 0.05). The mean corpuscular hemoglobin concentration (MCHC) was higher in the group of treated normal rats than in the other groups ( *p* < 0.05).

**Table 1:** Erythrogram of rats subjected to six weeks of regular exercise followed by maximal effort test

Variables	Without treatment		With treatment	
	
	Normal	Diabetic	Normal	Diabetic
Hemoglobin (g/dL)	12.67 ± 1.62^a^	12.63 ± 1.15^a^	14.61 ± 0.80^b^	14.61 ± 0.83^b^
Erythrocytes (10^6^/µl)	8.09 ± 0.19^a^	7.78 ± 0.45^a^	8.85 ± 0.44^b^	8.69 ± 0.40^b^
Hematocrit (%)	45.50 ± 3.59^a^	44.85 ± 4.40^a^	51.32 ± 3.07^b^	51.77 ± 2.20^b^
MVC (µm^3^)	52.57 ± 1.71^a^	55.62 ± 2.38^a^	57.25 ± 2.18^b,c^	59.62 ± 3.50^c^
MCH (pg)	16.50 ± 0.38^a^	17.60 ± 0.55^a,b^	17.86 ± 0.67^b^	17.93 ± 0.44^b^
MCHC (%)	30.21 ± 0.50^a^	31.30 ± 0.91^a^	32.61 ± 0.84	30.70 ± 1.24^a^

Averages followed by same letter are not different (Tukey) at a significance level of 0.05. MCV: mean corpuscular volume; MCH: mean corpuscular hemoglobin; MCHC: mean corpuscular hemoglobin concentration.

Table 2 presents the results of leukocyte counts in different groups. The treated groups showed higher values of leukocytes in relation to the untreated animals ( *p* < 0.05). But no difference was observed between diabetic and normal groups in both the absence and in presence of treatment with anabolic ( *p* > 0.05). In relation to total neutrophils, the DT group had the highest count among all groups studied ( *p* < 0.05). The situation was reversed in the count of lymphocytes, since it was lower in the DT group compared with group N ( *p* < 0.05). The values of band neutrophils were lower in DT than in D ( *p* < 0.05). The segmented neutrophils were higher only in the DT when compared with group N ( *p* < 0.05). Regarding eosinophils there was no difference between groups ( *p* > 0.05). For monocytes, the counts were lower in the DT group in comparison with D ( *p* < 0.05).

**Table 2:** Leucogram of Wistar rats submitted to six weeks of regular exercise followed by maximal effort test

Variables	Without treatment		With treatment	
	
	Normal	Diabetic	Normal	Diabetic
Leukocytes (cells/µl)	4100.0 ± 486.5^a^	4612.5 ± 458.0^a^	6025.0 ± 384.5^b^	6000.0 ± 1192.0^b^
Total neutrophils (%)	37.6 ± 7.7^a^	45.8 ± 11.15^a^	41.3 ± 7.0^a^	52.9 ± 5.2
Band neutrophils (%)	2.9 ± 0.9^a,b^	7.0 ± 5.6^a^	2,0 ± 1.4^b^	2.51 ± 0.9^b^
Segmented neutrophils (%)	34.7 ± 7.4^b^	38.8 ± 12.0^a,b^	39.3 ± 7.7^a,b^	50.4 ± 5.5^a^
Lymphocytes (%)	58.6 ± 5.4^b^	46.1 ± 12.1^a^	55.6 ± 5.6^a,b^	47.5 ± 5.1^a^
Eosinophils (%)	1.6 ± 0.5^a^	0.8 ± 0.7^a^	1.4 ± 0.5^a^	1.3 ± 0.7^a^
Basophils	0	0	0	0
Monocytes (%)	5.6 ± 1.5^a,b^	6.9 ± 3.9^a^	5.8 ± 3.5^a,b^	2.2 ± 2.0^b^

Averages followed by same letter are not different (Tukey) at a significance level of 0.05.

**Figure 1:**
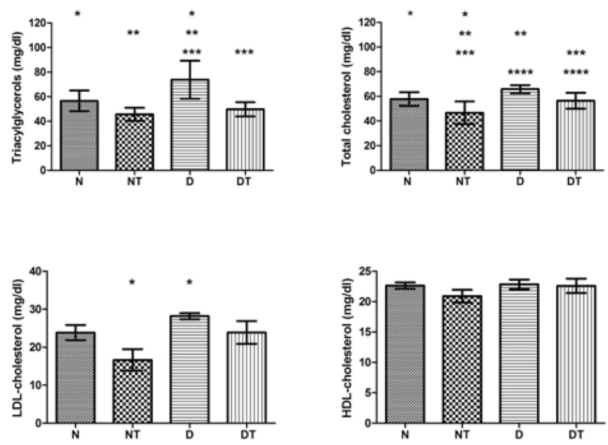
Plasma concentrations of triacylglycerols, total cholesterol, LDL-cholesterol and HDL-cholesterol after the maximal exercise test. The symbols N, NT, D and DT represent normal, treated normal, diabetic and treated diabetic rats, respectively. Values with the same symbol are significantly different (p < 0.05) when compared by Tukey test.

Figure 1 shows the results obtained in lipidogram of rats of different groups. The concentrations of triacylglycerols (TAG) were significantly higher in group D than in all the other groups (N, NT and DT), for a ( *p* < 0.05). The concentration of total cholesterol was higher in group D in relation to groups treated with the anabolic (DT and NT), for a ( *p* < 0.05), but not in relation to the healthy rats without treatment (N), for a ( *p* > 0.05). The values of HDL-C showed no changes resulting from physical training and treatment with testosterone ( *p* > 0.05). The concentration of LDL-C was higher in group D only in relation to group NT ( *p* < 0.05).

**Figure 2:**
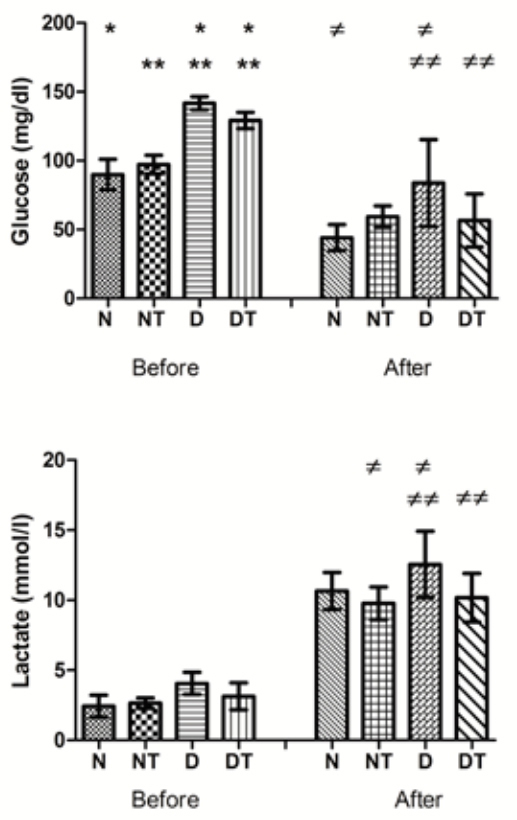
Plasma concentrations of glucose and lactate before and after the maximal exercise test. The symbols N, NT, D and DT represent normal, treated normal, diabetic and treated diabetic rats, respectively. Values with the same symbol are significantly different (p < 0.05) when compared by Tukey test.

Figure 2 shows the values of glycemia and lactatemia before and after the test of maximal effort for rats of different groups. Before the test, blood glucose was higher in the D group, followed by the DT group ( *p* < 0.05). The blood glucose values dropped significantly after the maximal effort test in all experimental groups, but still remained higher in group D ( *p* < 0.05).

Regarding lactatemia, there was no difference between groups before the effort test ( Figure 2 ), for a ( *p* > 0.05). After the test of maximum effort, the blood lactate concentration increased in all groups ( *p* < 0.05). It increased more in group D compared to groups NT and DT ( *p* < 0.05).

**Figure 3:**
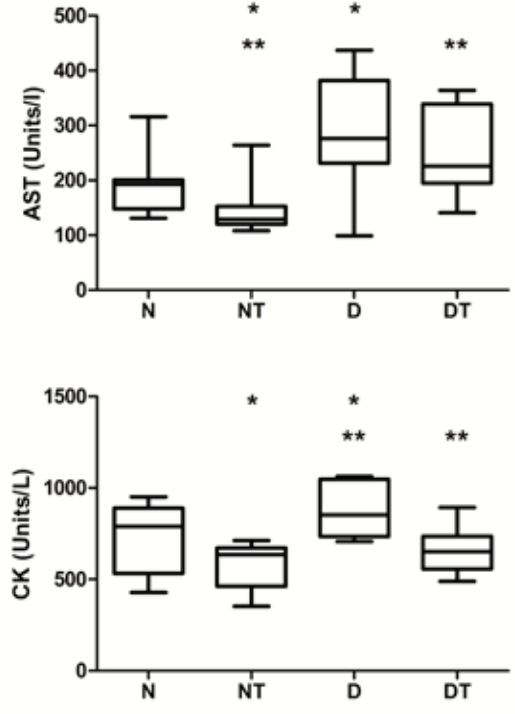
Plasma levels of activities of the enzymes aspartate aminotransferase (AST) and creatine kinase (CK) after the maximal exercise test. The symbols N, NT, D and DT represent normal, treated normal, diabetic and treated diabetic rats, respectively. Values with the same symbol are significantly different (p < 0.05) when compared by Tukey test.

Figure 3 shows the activity values of AST and CK after the effort test in all experimental groups. The activity of AST was lower in animals of group NT compared to animals in groups D and DT. The activity of CK was higher in group D when compared to groups NT and DT.

Table 3 shows time of forced swim and weight of the visceral fat in rats of different groups. Animals treated with testosterone (NT and DT) had longer times of forced swimming than their respective non-treated controls (N and D), for a ( *p* < 0.05). The amount of visceral fat was lower in groups treated with anabolic (NT and DT) in relation to their respective untreated controls (N and D), for a ( *p* < 0.05).

**Table 3:** Swimming time and mass of visceral fat of rats submitted to six weeks of regular exercise followed by maximal effort test

Parameter	Without treatment		With treatment	
	
	Normal (n = 8)	Diabetic (n = 8)	Normal (n = 8)	Diabetic (n = 8)
Swimming time (min)	203.75 ± 71.92	119.16 ± 55.34	305.80 ± 34.36	163.80 ± 67.51
Visceral fat (g)	4.22 ± 1.84 (1.34%)	2.27 ± 1.72 (0.69%)	1.68 ± 0.93^a^ (0.55%)	1.57 ± 0.65^a^ (0.48%)

Averages followed by same letter are not different (Tukey) at a significance level of 0.05. Values between parentheses represent the percentage of visceral fat in relation to the body mass.

## DISCUSSION

In the conditions considered in this study, treatment with testosterone produced an increase in the time of forced swimming in normal and diabetic rats in relation to their respective untreated controls. The hematological and biochemical records presented in this work should probably reflect some of the reasons that led to the change in performance of animals and any problems associated with treatment.

The decrease of CK in the diabetic rats treated with testosterone ( Figure 3 ) seems a remarkable fact, since elevations in blood activities of CK and AST from the liver have been reported in athletes using anabolic steroids ( 17 , 18 ) and even in athletes who do not use these substances ( 19 ). It seems reasonable to admit, then, that the anabolic might have increased the resistance of skeletal muscle fibers against injury in the diabetic group.

Although elevation in CK had been also attributed to increased permeability of the sarcolemma during exercise ( 20 , 21 ), the activity levels of serum CK are good markers of muscle injury ( 22 ). The blood levels of CK are associated to the intensity of exercise when duration is kept constant ( 20 ). The metabolic stress and the actions of contraction and relaxation can cause mechanical stress to the point of damaging the muscle tissue ( 23 ). In fact, a suitable training program for the physical fitness should not lead to sharp increase in the activities of AST and CK in plasma or serum ( 24 ).

The swimming by longer time in the treated group could also be due to increased availability of fatty acids in plasma, due to the mobilization of fat stores, since visceral fat actually fell in the animals treated with the anabolic. Testosterone might be associated with reduction in visceral fat content ( 25 , 26 ), since that aged rats have more visceral fat and produce less testosterone.

The metabolism of fatty acids involves higher consumption of oxygen than metabolism of glycogen associated with anaerobic glycolysis. It is well known that an increased necessity of oxygen stimulates the release of erythropoietin and, consequently, the production of red blood cells. Indeed, the quantity of erythrocytes, the concentration of hemoglobin and hematocrit values were higher in treated animals. In fact, it was reported that testosterone causes increased erythropoiesis in mice ( 27 ). This improvement in the erythrogram of the treated group would favor the consumption of oxygen and would increase the energetic efficiency of the aerobic catabolism. Indeed, after the exhaustion test, the amount of lactate in the blood of the diabetic rats was lower in the treated group than in non-treated ( Figure 2 ). This indicates that use of anabolic steroid associated to physical training was effective in aerobic conditioning of the animals.

Furthermore, the use of anabolic was associated with reduction in blood glucose, especially in the diabetic group ( Figure 2 ), as would be desirable. This could be due to the action of testosterone in the increase of Glut4 transcription and enzymatic activities of hexokinase and phosphofructokinase ( 28 ).

Further evidence of better utilization of fat induced by treatment with testosterone is a decrease in blood levels of TAG in diabetic rats ( Figure 1 ). The use of steroids combined with the practice of exercise increases the demand for fatty acids in energy metabolism, which certainly must be the factor responsible for decreased levels of TAG in this work.

The concentration of total cholesterol decreased significantly in healthy animals and in diabetic animals treated with testosterone, without association with changes in levels of HDL-cholesterol ( Figure 1 ), what agrees with the results reported by ( 29 , 30 ). Unlike that article, however, there was no decrease in levels of LDL-cholesterol in our work.

Some differences between the responses associated with our experimental design in relation to the literature should be the result of the complexity of the system. Undoubtedly, there must be differences and antagonisms in the results of the combination of time, nature and intensity of exercises with the nature, dose and duration of treatment with anabolic steroids.

Although the acute exercise is associated with elevated levels of cortisol and catecholamines, which would decrease the amount of leukocytes ( 31 , 33 ), and predominantly with elevation in the blood levels of white cells ( 34 ), studies in rats with alloxan-induced diabetes have shown that physical exercise causes changes in the immune system and decrease in serum glucose ( 35 , 36 ).

In our study the combination of anabolic with the program of exercise, followed by the exhaustion test, was associated with increased number of leukocytes in healthy and diabetic rats ( Table 2 ). This change should not be a consequence of the exhaustion test, but certainly of the use of anabolic, because the levels of leucocytes were higher in the groups treated with testosterone. These alterations could be due to decrease of oxygen in the blood or increase in mitotic activity of bone marrow cells, which may reflect exacerbation of the inflammatory processes, especially in the diabetic rats ( 29 , 37 ).

In conclusion, the combination of the use of testosterone with physical training during four weeks, followed by maximal effort test, produced elevations of hemoglobin, hematocrit and erythrocytes in healthy elderly rats and in elderly rats with alloxan-induced diabetes. Although these elevations had been greater in normal rats, they were associated with improvement in aerobic metabolism of fatty acids and glucose in diabetic animals. The hematological and biochemical changes were also associated with improvement in physiological characteristics, with increase of the swimming time and decrease of visceral fat levels in normal and diabetic animals. However, treatment with testosterone produced leukogram changes that may indicate exacerbation of inflammatory processes, especially among diabetic rats.
